# A Novel Hybrid Technique for Detecting and Classifying Hyperspectral Images of Tomato Fungal Diseases Based on Deep Feature Extraction and Manhattan Distance

**DOI:** 10.3390/s25144285

**Published:** 2025-07-09

**Authors:** Guifu Ma, Seyed Mohamad Javidan, Yiannis Ampatzidis, Zhao Zhang

**Affiliations:** 1 Research Institute of Agricultural Mechanization, Xinjiang Academy of Agricultural Sciences, Urumqi 830091, China; mgf1437@163.com; 2 Department of Biosystems Engineering, Tarbiat Modares University, Tehran 14115-111, Iran; 3 Agricultural and Biological Engineering Department, Southwest Florida Research and Education Center, University of Florida, 2685 FL-29, Immokalee, FL 34142, USA; 4 Key Laboratory of Smart Agriculture System Integration, Ministry of Education, China Agricultural University, Beijing 100083, China; zhaozhangcau@cau.edu.cn

**Keywords:** deep feature extraction, early disease detection, hyperspectral images, one-shot and few-shot learning, precision agriculture, tomato fungal diseases

## Abstract

Accurate and early detection of plant diseases is essential for effective management and the advancement of sustainable smart agriculture. However, building large annotated datasets for disease classification is often costly and time-consuming, requiring expert input. To address this challenge, this study explores the integration of few-shot learning with hyperspectral imaging to detect four major fungal diseases in tomato plants: *Alternaria alternata*, *Alternaria solani*, *Botrytis cinerea*, and *Fusarium oxysporum*. Following inoculation, hyperspectral images were captured every other day from Day 1 to Day 7 post inoculation. The proposed hybrid method includes three main steps: (1) preprocessing of hyperspectral image cubes, (2) deep feature extraction using the EfficientNet model, and (3) classification using Manhattan distance within a few-shot learning framework. This combination leverages the strengths of both spectral imaging and deep learning for robust detection with minimal data. The few-shot learning approach achieved high detection accuracies of 85.73%, 80.05%, 90.33%, and 82.09% for *A. alternata*, *A. solani*, *B. cinerea*, and *F. oxysporum*, respectively, based on data collected on Day 7 post inoculation using only three training images per class. Accuracy improved over time, reflecting the progressive nature of symptom development and the model’s adaptability with limited data. Notably, *A. alternata* and *B. cinerea* were reliably detected by Day 3, while *A. solani* and *F. oxysporum* reached dependable detection levels by Day 5. Routine visual assessments showed that *A. alternata* and *B. cinerea* developed visible symptoms by Day 5, whereas *A. solani* and *F. oxysporum* remained asymptomatic until Day 7. The model’s ability to detect infections up to two days before visual symptoms emerged highlights its value for pre-symptomatic diagnosis. These findings support the use of few-shot learning and hyperspectral imaging for early, accurate disease detection, offering a practical solution for precision agriculture and timely intervention.

## 1. Introduction

Tomatoes are among the most widely cultivated and economically important crops worldwide. However, their production is frequently threatened by fungal diseases that significantly affect both yield and fruit quality. Four major pathogens, *Alternaria alternata*, *Alternaria solani*, *Botrytis cinerea*, and *Fusarium oxysporum*, are particularly destructive, infecting various parts of the plant such as leaves, stems, and fruits. These diseases disrupt essential physiological processes, particularly photosynthesis, through chlorophyll degradation and pigment imbalances, resulting in symptoms like necrotic lesions, wilting, and discoloration [1]. Additionally, these infections impact other pigments like carotenoids and anthocyanins, further contributing to tissue damage.

Given the significant impact of these diseases on crop health and productivity, early and precise detection is critical for effective management and prevention. Traditional diagnostic methods such as visual inspections are fast and accessible but lack precision, often failing to distinguish between similar symptoms caused by different stressors. Laboratory-based diagnostics (e.g., fungal culturing, microscopy, and polymerase chain reaction -PCR) offer higher accuracy but are labor-intensive, time-consuming, and costly, limiting their scalability in large-scale farming operations [2,3]. As a result, there is a growing need for rapid, cost-effective, and scalable diagnostic solutions.

In recent years, researchers have successfully leveraged image processing and artificial intelligence (AI) techniques to detect and classify various plant diseases. These advancements have enabled the development of accurate, efficient, and scalable solutions for managing crop health and improving agricultural productivity. Several studies have demonstrated the effectiveness of deep learning models, particularly convolutional neural networks (CNNs), in classifying tomato diseases using large RGB image datasets. For example, models combining CNNs and Support Vector Machines (SVMs) trained on the PlantVillage dataset, which contained 18,835 RGB images, achieved up to 96.99% accuracy for tomato disease detection [4]. Ensemble approaches integrating Decision Trees and SVMs reached 95.98% accuracy on classifying tomato diseases [5], while ResNet-50-based models improved lesion localization and disease severity assessment with classification accuracy as high as 98.25% [6]. Other studies explored a range of deep learning architectures, including GoogleNet, AlexNet, and Inception v3, achieving accuracies between 65.8% and 96%, with ensemble voting methods pushing performance to nearly 100% [7,8]. These efforts highlight the potential of deep learning in tomato disease detection and management, though most rely on large, labeled RGB datasets and do not address early detection or data scarcity challenges.

Advances in hyperspectral imaging (HSI) and machine learning have opened new avenues for plant disease detection. HSI captures detailed spectral signatures of plant tissues, revealing subtle biochemical changes before visible symptoms appear. These spectral patterns can be analyzed by deep learning models for early, accurate, and non-invasive disease diagnosis. However, building such models typically requires large labeled datasets and expert-driven feature engineering, which might not be cost-effective and impractical in real-world agricultural settings [9].

Few-shot learning addresses these limitations by enabling model training with minimal annotated data. By leveraging deep learning to extract robust feature vectors from hyperspectral images, this approach eliminates the necessity for manual feature extraction by experts, significantly enhancing efficiency and reducing time and effort. The integration of the Manhattan distance method further enhances classification efficiency by measuring the resemblance between extracted features, allowing for precise differentiation between fungal diseases with limited data [10].

While previous studies have demonstrated high classification accuracy using CNNs and ensemble learning models on large RGB or hyperspectral image datasets, few have explored the integration of few-shot learning with HSI for early disease detection. In this study, a hybrid approach is proposed that combines HSI with EfficientNet-based deep feature extraction and Manhattan distance-based classification. This framework allows for precise differentiation between fungal diseases using only a few images per class, eliminating the need for large datasets or manual feature selection. This approach not only detects diseases accurately with minimal training data but also tracks disease progression by analyzing spectral changes over time. This capability enables early detection, ensuring timely intervention and reducing disease spread.

The novelty of this work lies in its application of few-shot learning to hyperspectral data and integrating it with the Manhattan distance metric for early-stage detection of multiple fungal diseases in tomatoes. By leveraging deep spectral features and a lightweight classification strategy, this study introduces an efficient, scalable, and cost-effective solution with real-time potential for precision agriculture, especially when integrated with Internet of Things (IoT)-enabled field systems.

## 2. Materials and Methods

**Figure 1:**
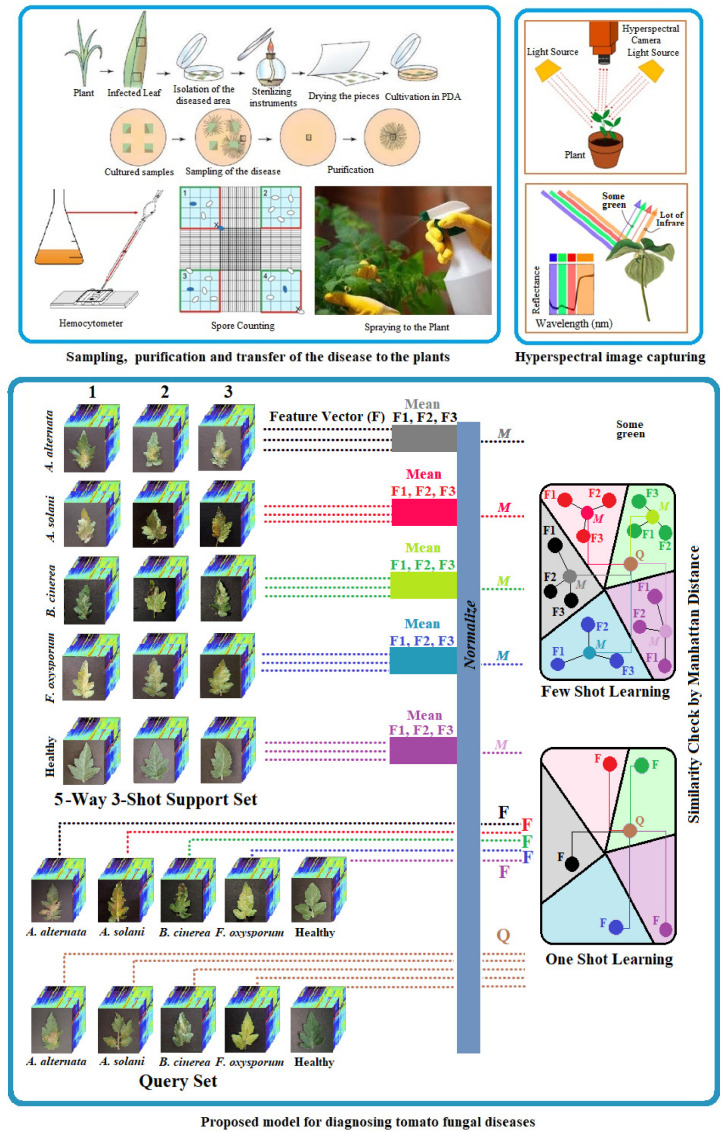
Workflow of the proposed framework.

This study focuses on the classification of tomato diseases caused by *A. alternata*, *A. solani*, *B. cinerea*, and *F. oxysporum*. After transplanting the pathogens to tomato seedlings, hyperspectral images of tomato leaves were collected every other day, starting from day 1 up to 7 days post inoculation. To ensure experimental control and enable accurate differentiation, images were also collected from a healthy control group, representing the disease-free class. The study employed one-shot learning (5-way 1-shot learning) and few-shot learning (5-way 3-shot learning) approaches to address the challenges associated with limited training data in classification tasks. EfficientNet was utilized for deep feature extraction due to its scalable and efficient architecture. Features extracted from input images were used to compute similarity measures based on the Manhattan distance method, facilitating accurate classification even in low-data scenarios. The workflow included dataset preparation, feature extraction, similarity calculation, and classification (Figure 1). These methods demonstrated the potential to achieve high performance in scenarios where labeled data is scarce.

### 2.1. Plant Material and Disease Induction

Tomato plants used in this study were cultivated in an experimental greenhouse at Gorgan University of Agricultural Sciences and Natural Resources, Iran. Seeds of *Solanum lycopersicum* (variety 4921) were surface sterilized using 1% sodium hypochlorite for 60 s and then sown in seedling trays filled with an equal mixture of coco peat, perlite, sand, field soil, and peat moss. After one month, seedlings were transferred into 1 kg pots and maintained in controlled greenhouse conditions (80% humidity, 20 ± 2 °C temperature). To ensure consistency and reliability in the experimental setup, a total of 40 tomato plants were cultivated and divided equally across four fungal disease classes and healthy controls (i.e., 8 infected and 2 healthy plants per class). Plants were grown under standardized environmental conditions, including consistent temperature, humidity, and lighting. These controlled conditions minimized environmental variability and ensured that the observed effects were solely due to fungal pathogens *A. alternata*, *A.solani*, *B. cinerea*, and *Fu. oxysporum*, and not to external factors. The fungal diseases were induced by inoculating the plants with spore suspensions of the respective pathogens, prepared at a concentration of 10^6^ spores/mL. This concentration was chosen to simulate natural infection conditions effectively while ensuring uniform exposure across all treated plants. A fine mist sprayer was used to evenly coat the surfaces of the leaves with the spore suspensions, ensuring that all parts of the plant were exposed to the pathogens. For the control group, the same procedure was followed using distilled water instead of spore suspensions, ensuring consistency in treatment across all plants while maintaining a disease-free baseline for comparison [11].

After inoculation, the plants were placed in an environment with high relative humidity (70%) for 48 h, a condition essential for fungal spore germination and successful infection. High humidity levels mimic conditions often found in tomato fields during disease outbreaks, promoting fungal growth and the onset of visible symptoms. Healthy plants that were not exposed to the spore suspensions were maintained as control samples. These control plants were kept under the same environmental conditions as the infected plants to ensure that differences observed during the experiment could be attributed directly to the infection and not to external variables. This systematic approach ensured uniformity in plant development and infection progression, providing a robust foundation for hyperspectral imaging, spectral analysis, and subsequent feature extraction and classification tasks.

### 2.2. Hyperspectral Image Acquisition

Hyperspectral images were collected every two days under controlled laboratory conditions using a Hyspim HSI-Vis_Nir-15fps spectroscopic camera (Hyspim, Stockholm, Sweden), starting immediately after the infection and continuing until seven days post inoculation. This systematic approach allowed for a detailed examination of disease progression and facilitated the identification of distinct spectral signatures corresponding to various stages of fungal infections in tomatoes. By capturing hyperspectral images at multiple time points, the dynamic biochemical and physiological changes in the leaves, driven by the infection, were effectively monitored over time.

The imaging system employed in this study operated within a spectral range of 400–950 nm, encompassing both the visible (VIS) and near-infrared (NIR) regions. This range was specifically chosen to capture critical reflectance features associated with chlorophyll content, water absorption, and other biochemical markers essential for assessing plant health and disease. To ensure data accuracy and consistency, rigorous calibration procedures were performed before each imaging session. Two primary calibration steps were implemented:

White Reference Calibration: A white reference panel with known reflectance properties was utilized to standardize the reflectance values in the images, compensating for variations in illumination and ensuring accurate representation of the optical properties of the leaf samples.

Dark Reference Calibration: A dark reference was recorded by blocking all incoming light to the imaging sensor, which corrected for baseline sensor noise and any signals unrelated to the sample’s reflectance [12].

Lighting consistency was a crucial aspect of the setup, achieved by using a halogen lamp as the light source. The halogen lamp provided stable, broad-spectrum illumination across the spectral range, ensuring even lighting over the leaf surface during imaging. To avoid shadows and glare, the halogen lamps were installed at a 45-degree angle relative to the samples, while the camera was positioned vertically (perpendicular) at a fixed distance of 25 cm from the leaf surface. This careful arrangement minimized shadows, glare, and environmental interferences, thereby enhancing the quality of the hyperspectral data. To simulate realistic variability while maintaining experimental control, healthy and infected leaves were excised from the plants at defined days post inoculation (1, 3, 5, and 7 days) and were imaged individually. During each imaging session, the leaves were intentionally placed at slightly different orientations and angles under the camera to mimic natural variability in leaf positioning as encountered in real-world conditions.

Additionally, a white reference sample was recorded every 10 min to recalibrate reflectance values and compensate for any fluctuations in light source irradiance during extended imaging sessions. The white reference was a matte white paper (non-reflective), measuring approximately 10 × 10 cm, and recommended by the hyperspectral camera manufacturer. This carefully designed imaging setup, incorporating calibrated measurements, consistent lighting, and periodic data acquisition, formed a robust foundation for analyzing the spectral characteristics of healthy and infected tomato leaves.

**Figure 2:**
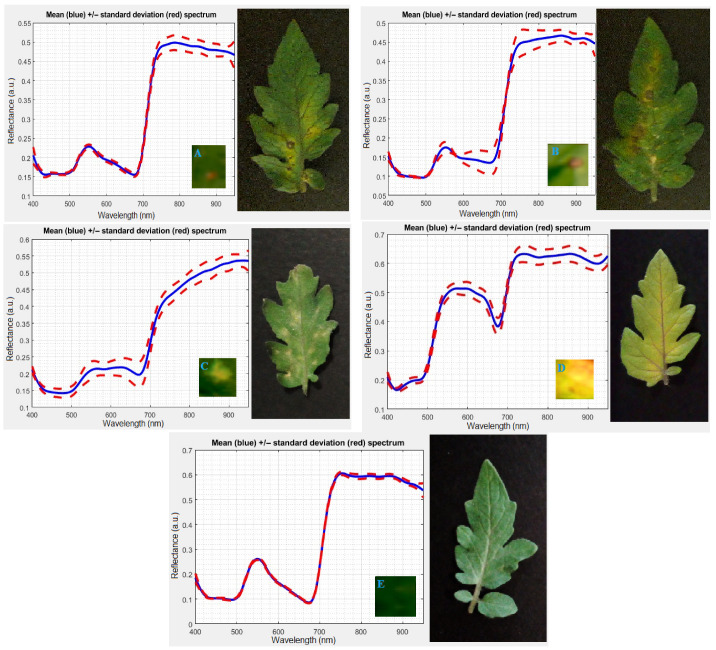
Representative RGB images and corresponding spectral signatures (400–950 nm) from symptomatic regions of tomato leaves infected with (**A**) *A. alternata*, (**B**) *A. solani*, (**C**) *B. cinerea*, (**D**) *F. oxysporum*, and (**E**) a healthy leaf.

The progression of visible symptoms for each disease was closely monitored through daily visual inspections conducted under consistent laboratory lighting conditions. This was done in parallel with hyperspectral image acquisition to establish a reference point for when symptoms became observable to the naked eye. For *A. alternata* and *B. cinerea*, the first visible signs of infection, such as small necrotic spots or localized discoloration, were recorded on Day 5 post inoculation. In contrast, plants infected with *A. solani* and *F. oxysporum* did not exhibit any visible symptoms until Day 7. No disease symptoms were observed in the control plants throughout the duration of the experiment. These observations provided a crucial baseline for evaluating the ability of the hyperspectral imaging system and AI-enabled technique to detect diseases prior to the manifestation of physical symptoms, thereby offering a non-invasive approach for early detection that precedes traditional visual diagnosis methods. Figure 2 presents representative RGB images alongside spectral signatures from the regions of interest (ROIs) for the four tomato leaf diseases examined, as well as a healthy leaf, spanning the full hyperspectral range of 400 to 950 nm. These images and spectral data were obtained from leaves exhibiting visible symptoms, with ROIs carefully selected from the symptomatic areas to capture disease-specific spectral characteristics.

### 2.3. Feature Extraction with EfficientNet

The EfficientNet is a well-established convolutional neural network (CNN) architecture designed for optimized feature extraction in image classification and computer vision tasks. Unlike traditional scaling methods that arbitrarily increase network depth, width, or input resolution, EfficientNet employs a compound scaling strategy, which balances all three dimensions in a principled and efficient manner. This approach allows the network to adapt flexibly to various resource constraints while maintaining high performance [13].

The compound scaling method in EfficientNet is based on the observation that depth, width, and input resolution are interdependent. Higher resolution images require deeper networks to capture large-scale features and wider networks to retain fine-grained details. By scaling these dimensions using specific ratios, EfficientNet achieves superior accuracy with fewer computational resources compared to conventional CNN architectures [14].

#### 2.3.1. Feature Extraction Process

**Figure 3:**
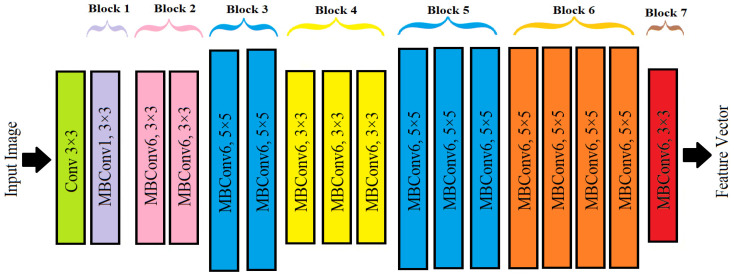
Architecture of the EfficientNet deep learning model used for hyperspectral feature extraction.

Figure 3 shows an architecture for feature extraction using EfficientNet deep learning. The feature extraction process in EfficientNet begins with image preprocessing, where each input image corresponds to a single spectral band extracted from the hyperspectral cube, which is resized to a fixed resolution (typically 224 × 224 pixels) and normalized. Since EfficientNet requires 3-channel inputs, each single-band image was converted to a 3-channel grayscale image by replicating the band across all three channels. No modification was made to the original EfficientNet architecture. A pre-trained EfficientNet model (trained on ImageNet) was used in feature extraction mode, where all weights and biases were kept frozen. The extracted features were not fine-tuned but directly used for computing similarity via the Manhattan distance metric for classification. The preprocessed image is then passed through a series of convolutional layers that progressively extract hierarchical features [15].

A key component of EfficientNet is the MBConv block (Mobile Inverted Bottleneck Convolution), which is derived from MobileNetV2. This block enhances feature extraction efficiency by incorporating depthwise separable convolutions, reducing computational cost while maintaining rich feature representations. Additionally, squeeze-and-excitation (SE) optimization is applied within these blocks to emphasize essential features and suppress less informative ones, further improving model performance. Following the convolutional layers, feature maps undergo pooling operations, such as max pooling or average pooling, to reduce spatial dimensions while preserving critical information. The final step involves flattening the feature maps and passing them through a fully connected layer to generate a compact feature vector. This feature vector was not used in a trainable classifier but instead served as an input for a similarity-based classification using Manhattan distance [14,16].

#### 2.3.2. Advantages of EfficientNet for Feature Extraction

By balancing depth, width, and resolution, EfficientNet achieves higher accuracy with fewer parameters and lower computational requirements. The compound scaling method enables seamless adaptation to different computational budgets, making it ideal for resource-constrained environments. Furthermore, the use of MBConv blocks and squeeze-and-excitation mechanisms ensures that only the most relevant features are extracted, improving the robustness of downstream tasks. Although various CNN architectures can be employed for feature extraction, EfficientNet has been chosen in this work due to its documented balance between accuracy and efficiency in prior literature. The architecture is considered particularly appropriate for applications requiring compact models with high performance, such as hyperspectral image analysis. However, it is recognized that model effectiveness is also influenced by the characteristics of the dataset and experimental configuration [15]. Recent studies have demonstrated the effectiveness of advanced CNN architectures such as ResNet-50 and EfficientNet in plant disease classification tasks. While traditional CNNs offer a solid foundation, their limited interpretability and scalability pose challenges for real-world agricultural applications. To overcome these limitations, researchers have incorporated attention mechanisms and optimized network structures to focus on disease-relevant image regions, thereby improving both accuracy and interpretability. For example, experiments on the PlantVillage dataset showed that basic CNNs achieved only 46.69% accuracy, while ResNet-50 and EfficientNet significantly outperformed them with 63.79% and 98.27% accuracy, respectively [16]. In another study focused on apple leaf disease classification, EfficientNet was selected for its optimal balance of performance and computational efficiency. A modified version of EfficientNetB0, tailored for resource-constrained environments, achieved training, validation, and testing accuracies of 99.10%, 97.40%, and 84.50%, respectively, while reducing the parameter count by up to 50% [17]. These results further support our selection of EfficientNet for hyperspectral image feature extraction, particularly in agricultural scenarios where both high accuracy and low computational cost are essential. Although EfficientNet is not a newly introduced model, its proven adaptability and efficiency remain relevant, especially when integrated into few-shot learning frameworks for complex image analysis tasks.

### 2.4. Data Normalization

Normalization is a fundamental technique in the data preprocessing stage of data mining. It involves transforming data from its original range into a new range, typically within a specified interval. This method plays a crucial role in enhancing the effectiveness of predictive modeling and analysis, as it aligns data for various forecasting models. By reducing discrepancies among diverse forecasting outputs, normalization ensures consistency and comparability across models.

Data normalization also enhances the precision and efficiency of machine learning models. It is particularly beneficial for algorithms that rely on distance metrics, as it prevents features with larger numerical values from disproportionately influencing the learning process. Among the various normalization methods, min–max normalization (commonly referred to as rescaling) is one of the simplest and most widely used [9]. This approach scales the data to a defined range, such as [0, 1] or [−1, 1], based on Equation (1).

(1) $$X_{\mathrm{norm}}=\frac{X-X_{\min}}{X_{\max}-X_{\min}}$$

where X_norm_ is the normalized data, X represents the original data, and X_max_ and X_min_ denote the maximum and minimum values of the data, respectively.

It is important to note that in few-shot learning, data must first be averaged before applying normalization. However, this preliminary step is not required in the case of one-shot learning, where the normalization process can be applied directly.

### 2.5. Few-Shot Learning

Few-shot learning is particularly well suited for plant disease detection tasks using hyperspectral data, where collecting large numbers of labeled samples is both time-consuming and costly, especially in the early stages of disease when symptoms are not yet visible. Unlike traditional methods that rely heavily on data augmentation or extensive manual annotation, few-shot learning enables models to generalize from only a few labeled examples per class, making it ideal for real-world agricultural scenarios where data scarcity is a critical limitation.

In our framework, we adopt a prototypical few-shot learning approach to classify hyperspectral signatures of infected and healthy leaf samples. The few-shot model is trained using a support set (S) and a query set (Q), where each sample in the query set represents an unseen instance to be classified based on the information in the support set. This setup follows the standard N-way, K-shot format, where N is the number of classes (e.g., different disease types or healthy class) and K is the number of labeled samples per class, typically kept small to simulate real-world constraints.

To extract meaningful spectral features, we use a feature extraction function f_θ_, which transforms the raw hyperspectral inputs into lower-dimensional embeddings. For each class, a prototype is computed by averaging the feature vectors of its K support samples. For each category, a class prototype is derived by computing the mean feature representation of the respective support set samples. To classify a new query sample x∈Q, a similarity metric is used to assess the relationship between the query sample and each class prototype. The similarity function, denoted as sim (⋅), compares the feature representations of the query sample with those of the prototypes. The obtained similarity values are then normalized to a range between 0 and 1 through a scaling transformation. Finally, these scaled similarity scores are fed into a linear function to determine the predicted class label for the query sample. This process is mathematically represented as Equation (2).

(2) $$P(y=k\left|x)\right.=\frac{{\exp(\mathrm{sim}(f}_{\theta }{(x),c}_{k})\;t}{\sum_{j=i}^{N}{\exp(\mathrm{sim}(f}_{\theta }{(x),c}_{j})\;t}$$

where x∈Q represents the query sample, c_k_ denotes the class prototype, N is the total number of categories in the few-shot learning task, and T is the scaling transformation applied to the similarity scores.

The prototypes, as representatives of each class, play a crucial role in the classification process, making few-shot learning a robust and efficient technique for scenarios with limited labeled data.

### 2.6. Similarity Measure Based on Manhattan Distance After Feature Extraction by EfficientNet

In our framework, deep features are extracted from hyperspectral image samples using an EfficientNet architecture tailored for spectral data. To perform classification within the few-shot learning setup, it is necessary to quantify the similarity between the feature representation of a query sample and the class prototypes derived from the support set.

We use Manhattan distance as the similarity measure. This choice is motivated by its robustness in high-dimensional spaces and its effectiveness in capturing subtle variations in hyperspectral features, critical for detecting early-stage plant diseases where class differences are subtle. The Manhattan distance computes the sum of absolute differences between the corresponding elements of two feature vectors, offering a straightforward and interpretable similarity metric.

This approach aligns well with our experimental goal: identifying early biochemical changes in plant tissue from limited data. The Manhattan distance’s emphasis on individual feature differences complements the nature of hyperspectral signals, where small shifts across narrow spectral bands can indicate disease onset. Moreover, we empirically found it to outperform more complex similarity functions in our few-shot classification setting. By specifying and justifying this choice, we aim to support both the scientific rationale and reproducibility of our method for researchers working with limited hyperspectral data in agricultural applications.

#### 2.6.1. EfficientNet Feature Vectors

EfficientNet generates feature representations f(x) for each input sample x. These features, often high-dimensional vectors, encode the essential characteristics of the input data. f(x) is the feature vector of a sample x, extracted by the EfficientNet model, and f(y) is the feature vector of another sample y.

#### 2.6.2. Manhattan Distance

The Manhattan distance d(x,y) between two feature vectors f(x) and f(y) is defined as the sum of the absolute differences between their corresponding components. This distance is calculated by Equation (3) [18].

(3) $$d(x,y)=\sum_{i=1}^{n}\left|f_{i}\left(x\right)-f_{i}\left(y\right)\right|$$

where n is the number of dimensions in the feature vector, f_i_(x) is the i-th component of the feature vector for sample x, and f_i_(y) is the i-th component of the feature vector for sample y.

#### 2.6.3. Use in Few-Shot Learning

In a few-shot learning scenario, the Manhattan distance is used to compare the query sample’s feature vector f(x) with the prototypes c_k_ of each class. The class prototype c_k_ is typically the mean of feature vectors in the support set for that class (Equation (4)) [18].

(4) $$c_{k}=\frac{1}{k}\sum_{j=1}^{k}{f(s}_{j})$$

where s_j_ is the j-th sample in the support set for class k, and K is the number of samples per class in the support set. To classify the query sample x∈Q, the Manhattan distance between f(x) and each prototype c_k_ is calculated.

#### 2.6.4. Decision Rule

The query sample x is assigned to the class k^∗^ with the smallest Manhattan distance (Equation (5)) [17,18].

(5) $$k*=\arg\;\min\;{d(x,c}_{k})$$

In this study, two few-shot learning scenarios were implemented: 5-way 1-shot learning and 5-way 3-shot learning, each involving classification among four fungal disease classes (*A. alternata*, *A. solani*, *B. cinerea*, and *F. oxysporum*) and healthy leaves. For each class in the 5-way 1-shot setting, only one hyperspectral image was used to extract features, which constituted the support set. In contrast, in the 5-way 3-shot setting, three hyperspectral images per class were used for feature extraction. Features from these three images were individually extracted and normalized, then averaged to create a single representative feature vector for each class in the support set. Classification was performed by comparing the feature vector of a query image against the averaged support feature vectors for each class using the Manhattan distance metric. The class with the minimum distance was assigned as the predicted label.

To ensure class balance, an equal number of support images was used for all five classes in each episode. Support and query images were selected to be mutually exclusive, ensuring no overlap between them during each evaluation. Query images were randomly sampled from the remaining labeled dataset, and multiple episodes were generated to assess performance stability across varied combinations. Selection of support samples was performed manually to maintain consistent quality and disease stage representation.

To complement this classification framework, 5-fold cross-validation was employed to ensure robust and reliable performance evaluation in both the 1-shot and 3-shot learning scenarios. For imaging on each day of the study, in the 1-shot setting, 10 hyperspectral images were selected per disease class (50 in total), with one image per class used for training (support set) and a different one for testing (query set) in each fold. Similarly, in the 3-shot setting, 20 images per class (100 in total) were used, with three images for training and one for testing per class per episode. Across all folds, support and query samples remained mutually exclusive, and class balance was maintained. Final accuracy values were reported as the average across all folds and evaluation episodes, providing a stable estimate of the model’s generalization capability under limited-data conditions.

#### 2.6.5. Advantages of Manhattan Distance

Manhattan distance is effective due to its robustness to outliers and computational simplicity, as it calculates absolute differences and handles high-dimensional, sparse feature vectors well. Combined with EfficientNet’s feature extraction, it provides accurate and efficient similarity measurement for classification and related tasks [19]. In the few-shot learning setting, especially 1-shot, where only one support example per class exists, classification accuracy based on Manhattan distance between feature vectors was chosen as the main evaluation metric. This reflects the model’s ability to discriminate classes with limited data. Although metrics like precision, recall, and F1-score are useful for larger datasets, they offer limited insight in very low-data scenarios and were therefore excluded.

## 3. Results

### 3.1. Disease Diagnosis Performance Across Imaging Days Using 5-Way One-Shot Learning

**Figure 4:**
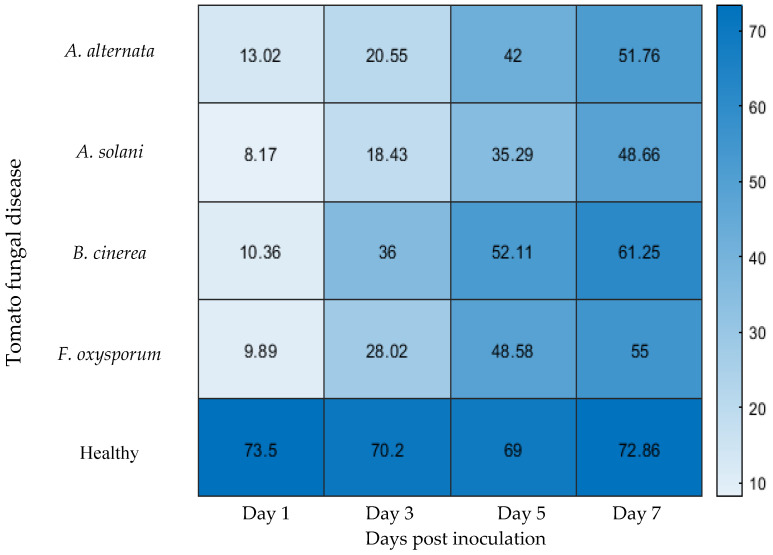
One-shot learning accuracy (%) for tomato fungal disease detection across different days post inoculation. Early visual symptoms of *A. alternata* and *B. cinerea* appeared on Day 5, and visual symptoms of *A. solani* and *F. oxysporum* on Day 7.

Figure 4 presents the results of One-Shot Learning for detecting tomato fungal diseases across different imaging days, demonstrating a notable increase in classification accuracy as the infection progresses. The recorded low accuracy results present the challenges of early detection, whereas later stages reveal clearer disease patterns that enable more reliable identification. Each fungal disease exhibits a consistent increase in classification accuracy over time, highlighting the growing distinctiveness of spectral signatures as the infection advances. Note that early visible symptoms of infection, such as small necrotic lesions or localized discoloration, were first observed on Day 5 post inoculation in plants infected with *A. alternata* and *B. cinerea*. In contrast, symptoms did not appear until Day 7 in plants inoculated with *A. solani* and *F. oxysporum*. Additionally, no disease symptoms were observed in the control plants (healthy leaves) at any point during the experimental period.

One observation from the results is the steady increase in classification accuracy from Day 1 to Day 7. Initially, the model struggles to achieve high accuracy due to the subtle spectral differences in the early stages of infection. On Day 1, accuracy is at its lowest, ranging from 8.17% (*A. solani*) to 13.02% (*A. alternata*). As the infection progresses, spectral variations become more pronounced, leading to improved classification performance. By Day 3, accuracy has increased across all diseases, with values ranging between 18.43% and 36%, indicating that early symptoms, though faint, are becoming detectable.

As the infection advances, accuracy improves considerably by Day 5, where the model achieves a range between 35.29% and 52.11%. At this stage, biochemical changes in the leaves, such as chlorophyll degradation and pigment alterations, become more evident, allowing hyperspectral imaging to capture stronger spectral features [20]. By Day 7, the classification accuracy reaches its peak, with values spanning 48.66% to 61.25%, indicating that the model performs significantly better once the disease symptoms fully manifest.

Among the four diseases analyzed, *B. cinerea* consistently exhibits the highest classification accuracy across all time points. By Day 3, its accuracy is already at 36%, and by Day 7, it reaches 61.25%, surpassing the detection rates of the other fungal pathogens. This suggests that *B. cinerea* produces distinct spectral changes that are easier to identify, even in the early phases of infection. It is possible that this pathogen causes more rapid biochemical alterations, making its spectral signature stand out earlier than the others [20].

In contrast, *A. solani* consistently registers the lowest accuracy rates throughout the experiment. On Day 1, its classification accuracy is only 8.17%, and by Day 7, it reaches 48.66%, which is still lower than the highest values recorded for other diseases. This trend suggests that *A. solani* exhibits subtle spectral differences in the early stages, making it more difficult for the model to distinguish. A potential explanation is that the physiological changes induced by this pathogen occur gradually, delaying the appearance of detectable spectral signatures.

Both *A. alternata* and *F. oxysporum* follow a similar trajectory, with their accuracy levels increasing at comparable rates. *A. alternata* starts at 13.02% and peaks at 51.76%, while *F. oxysporum* begins at 9.89% and reaches 55% on Day 7. These results indicate that while both diseases become easier to classify over time, *F. oxysporum* appears to have a slightly stronger spectral distinction at later stages. This may be attributed to the nature of the pigment degradation and biochemical disruptions caused by this pathogen.

The progressive increase in classification accuracy over time can be attributed to several physiological changes in infected leaves. One primary factor is the gradual breakdown of chlorophyll, which influences reflectance in the visible spectrum. In the initial stages of infection, chlorophyll levels remain relatively high, making it difficult to differentiate between healthy and diseased leaves. However, as the infection progresses, chlorophyll degradation becomes more evident, resulting in distinct spectral variations that improve classification accuracy. Additionally, moisture loss plays a significant role, particularly in the near-infrared spectrum. Infected leaves experience a steady decline in water content, altering their spectral reflectance in a way that becomes more distinguishable at later stages. Structural modifications, such as the development of lesions and necrotic areas, further enhance classification performance. These physical alterations change the way light interacts with the leaf surface, introducing new spectral features that the model can leverage for more accurate disease identification [21].

Another contributing factor to lower accuracy in early disease detection is the limited sample size inherent in One-Shot Learning. Because this approach relies on a single reference image per class for training, the model has minimal exposure to natural variations within each disease type. As a result, it struggles to generalize effectively, particularly in the early stages of infection when spectral differences are subtle. The lack of sufficient training samples reduces the model’s ability to distinguish between infected and healthy leaves, leading to lower classification performance [21].

A practical solution to this limitation is the implementation of Few-Shot Learning, which allows the model to learn from a slightly larger set of examples. This additional training data helps the model build more comprehensive feature representations, improving its ability to detect diseases even in the early stages.

### 3.2. Disease Diagnosis Performance Across Imaging Days Using 5-Way 3-Shot Learning

**Figure 5:**
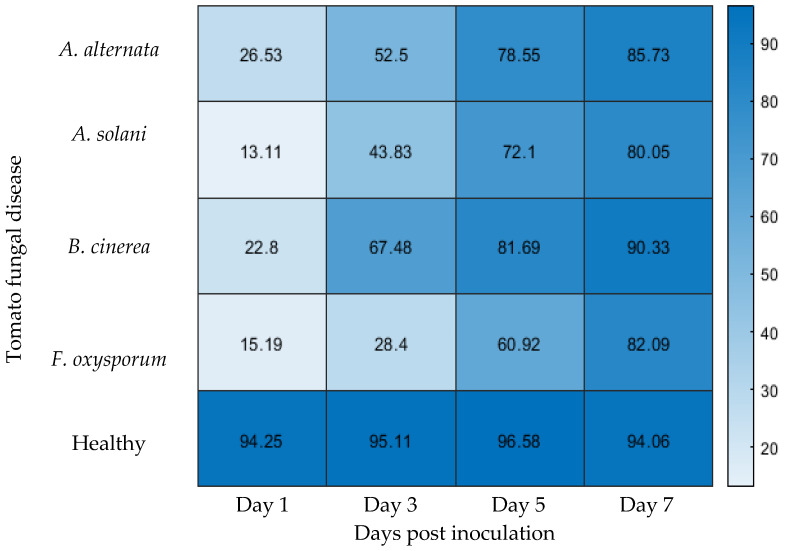
Few-shot learning accuracy (%) for tomato fungal disease detection across different days post inoculation. Early visual symptoms of *A. alternata* and *B. cinerea* appeared on Day 5, and visual symptoms of *A. solani* and *F. oxysporum* appeared on Day 7.

Figure 5 presents the classification accuracy for the four plant diseases (*A. alternata*, *A. solani*, *B. cinerea*, and *F. oxysporum*) and healthy leaves across different imaging dates, utilizing the few-shot learning approach. Hyperspectral images were collected on Days 1, 3, 5, and 7, showing a clear trend of improved model performance as more data becomes available. The proposed model demonstrated consistently high accuracy in detecting healthy (control) leaves across all imaging days. This strong performance is attributed to the consistency of the extracted feature vectors over time. Specifically, the classification accuracies for control plants on Days 1, 3, 5, and 7 following the application of distilled water were 94.25%, 95.11%, 96.58%, and 94.06%, respectively, yielding an average accuracy of 95%. These results highlight the model’s robustness in identifying healthy leaves throughout the experiment and confirm the reliability of the few-shot learning framework. However, the remaining 0.5% classification error indicates that, in rare cases, healthy plants may still be misclassified as diseased. In future applications, reducing this error will be critical to avoid unnecessary interventions. Therefore, integrating expert validation to cross-check model predictions would enhance the reliability and trustworthiness of the system in real-world deployment scenarios.

As expected in few-shot scenarios, Day 1 accuracies were relatively low due to limited training examples. However, with additional imaging days, the model demonstrated increasing accuracy, highlighting its capacity to learn and generalize disease features with minimal data [22].

Among the four diseases, *A. alternata* exhibits the most consistent and rapid improvement in detection accuracy. It starts at 26.53% on Day 1, increases significantly to 52.5% on Day 3, further rises to 78.55% on Day 5, and ultimately reaches 85.73% on Day 7. This steady progression reflects the model’s ability to gradually learn and identify key disease features over time. *B. cinerea* also shows a sharp improvement, beginning at 22.8% on Day 1, increasing to 67.48% by Day 3, reaching 81.69% on Day 5, and achieving the highest final accuracy of 90.33% on Day 7. The model’s strong performance with *B. cinerea* may be attributed to its distinct visual symptoms, which make it easier for the model to learn and classify earlier in the infection process.

In contrast, *A. solani* and *F. oxysporum* show comparatively slower improvements. *A. solani* starts with the lowest initial accuracy of 13.11% on Day 1, improving to 43.83% on Day 3, reaching 72.1% on Day 5, and culminating at 80.05% on Day 7. Similarly, *F. oxysporum* begins at 15.19% on Day 1, increases to 28.4% on Day 3, then progresses to 60.92% on Day 5, and ends at 82.09% on Day 7. The lower initial accuracy for these diseases may be due to more subtle symptom patterns, requiring more exposure to data for the model to effectively learn distinguishing features. However, by Day 7, both diseases achieve a high detection accuracy, indicating that with sufficient training examples, the model can successfully classify them.

The observed improvements in accuracy are primarily driven by two factors: the progression of disease symptoms over time and the increasing number of images available for training. As the days progress, the model is exposed to more images, enhancing its ability to generalize and recognize patterns. On Day 1, the model struggles with distinguishing between diseases due to the limited dataset and subtle early-stage symptoms. However, as more days of imaging are incorporated, the model gains access to additional training examples, which strengthens its ability to detect diseases more accurately. Simultaneously, as the diseases progress, their symptoms become more pronounced, making it easier for the model to identify characteristic spectral and visual features [23].

In conclusion, the data clearly indicates that disease detection accuracy improves over time with the inclusion of more training images and the natural progression of the diseases themselves. While some diseases, such as *B. cinerea*, exhibit a faster learning curve and achieve high accuracy quickly, others, like *A. solani* and *F. oxysporum*, require more time and exposure to data before reaching optimal performance. The combined effect of increasing training data and evolving disease symptoms leads to higher detection accuracy, highlighting the effectiveness of few-shot learning in plant disease identification when paired with an expanded dataset and the natural symptom development over time [24]. To assess the impact of different similarity metrics on classification performance, cosine similarity was employed as an alternative to Manhattan distance for measuring the similarity between feature vectors extracted from hyperspectral images using the EfficientNet architecture. As shown in Table 1, classification based on cosine similarity also produced competitive results; however, slightly lower accuracy was observed compared to the Manhattan distance. This outcome indicates that Manhattan distance is more effective in capturing subtle spectral variations among disease classes in hyperspectral data. The relatively lower performance of cosine similarity may be attributed to its sensitivity to the orientation of vectors rather than their absolute differences, which makes it less suited for distinguishing fine-grained intensity variations commonly present in hyperspectral feature representations.

### 3.3. Optimal Early Disease Detection Day

Understanding the timing of symptom onset is essential for evaluating the practical utility of non-invasive technologies for early disease diagnosis in plants. In this study, routine visual assessments revealed that infections by *A. alternata* and *B. cinerea* became visibly apparent only by Day 5 post inoculation, while *A. solani* and *F. oxysporum* did not exhibit any visible symptoms until Day 7. This lag between infection and visual symptom development represents a critical window during which conventional observation methods may fail to detect diseases. In contrast, AI-enabled hyperspectral imaging demonstrated the capability to identify subtle physiological changes much earlier, capturing minute spectral variations before any visible symptoms appeared. This early detection capability highlights the scientific innovation and practical value of hyperspectral imaging, reinforcing its potential as a powerful tool for pre-symptomatic disease detection in plant health monitoring.

To determine the optimal timing for early intervention, it is essential to identify the earliest day on which the model achieves sufficient accuracy to reliably distinguish between different diseases. Model performance was evaluated across multiple days to establish when each disease could first be detected with confidence. The following analysis highlights the earliest day for reliable detection, balancing both accuracy and timeliness for each pathogen.

*A. alternata*: The model’s accuracy was low on Day 1 (26.53%) but improved to 52.50% by Day 3. Since visual symptoms appeared on Day 5, the model demonstrated the ability to detect the disease two days before visible signs emerged, making Day 3 a viable point for early, pre-symptomatic intervention.

*A. solani*: Accuracy began at 13.11% on Day 1, improved to 43.83% on Day 3, and reached 72.10% by Day 5. Visual symptoms were not observed until Day 7, indicating that the model could reliably detect the disease two days earlier than traditional visual methods. Therefore, Day 5 is recommended for early, yet accurate, detection.

*B. cinerea*: Accuracy increased from 22.80% on Day 1 to 67.48% on Day 3, then further to 81.69% on Day 5, and 90.33% by Day 7. With visual symptoms first appearing on Day 5, the model provided a reliable signal two days prior on Day 3, supporting its use for pre-symptomatic detection.

*F. oxysporum*: Starting at 15.19% on Day 1, the model’s accuracy reached only 28.40% by Day 3, but improved to 60.92% by Day 5 and 82.09% by Day 7. Since symptoms were visible only from Day 7 onward, Day 5 offers the earliest point for confident detection, allowing a two-day lead over symptom-based observation.

In summary, the model demonstrates clear potential for pre-symptomatic disease detection. *A. alternata* and *B. cinerea* can be reliably identified by Day 3, two days before symptoms are visible. *A. solani* and *F. oxysporum*, while requiring until Day 5 for accurate classification, are still detected ahead of visual symptom onset, reinforcing the value of this approach for timely intervention and management.

## 4. Discussion

Timely detection and intervention in plant diseases are essential for minimizing economic losses. Traditionally, plant disease diagnosis has relied on manual inspection by experts, a method that is time-consuming, labor-intensive, and prone to human error. Recent advances have introduced AI and remote sensing technologies to enhance disease detection and classification [21]. Among these, deep learning approaches have gained significant attention, particularly those using RGB datasets such as PlantVillage [22,23,24]. Although these datasets effectively capture visual disease symptoms, real-world conditions often involve plants at various stages of infection, which complicates detection and reduces model accuracy [25]. To overcome data limitations, augmentation techniques, such as flipping, rotating, cropping, resizing, and adding noise, have been employed to increase dataset diversity and improve model generalization [26,27]. Furthermore, hybrid models that combine deep learning for feature extraction with machine learning for classification have shown potential [28]. Nevertheless, these methods still depend heavily on large labeled datasets, which are often costly and difficult to obtain in agricultural settings.

Hyperspectral imaging has emerged as a powerful tool due to its ability to capture fine-grained spectral information beyond standard RGB imaging [29,30,31]. This allows for early and accurate disease detection. Nonetheless, the high cost of hyperspectral sensors and the volume of data required for training models hinder large-scale deployment [32]. To address these challenges, researchers have investigated the use of machine learning, rather than deep learning, on hyperspectral data, as it requires fewer samples [33,34]. These models can achieve strong performance, but their success depends heavily on effective feature extraction and selection [35]. Identifying the most informative features demands expertise in data processing to ensure accuracy and robustness. Techniques such as Scale-Invariant Feature Transform (SIFT) have been used to extract key image features across different scales and conditions, enhancing model adaptability [36].

Another significant challenge is distinguishing diseases with similar visual symptoms. Several studies [10,37,38] have proposed advanced algorithms and segmentation techniques to address this issue. These deep learning-based segmentation methods can accurately locate and analyze diseased regions but often require substantial computational power and high-resolution imaging data. Weight-based learning techniques have also been explored to boost diagnostic precision by adjusting model parameters based on the importance of specific features [10,39]. Although highly accurate, these methods again require extensive labeled datasets, which are often difficult to gather in agricultural contexts. Few-shot learning has recently emerged as a promising solution, enabling disease recognition with minimal labeled data. This approach significantly enhances the speed, precision, and scalability of disease detection, making it a compelling alternative to traditional methods.

In this study, these limitations have been addressed by utilizing hyperspectral images with a restricted number of samples, demonstrating that accurate plant disease diagnosis can be achieved without the need for extensive datasets. By reducing dependence on large and expensive datasets, the feasibility and cost-effectiveness of hyperspectral imaging-based disease detection have been improved. Additionally, computational demands have been lowered, facilitating real-time, in-field applications and enabling faster disease identification and intervention.

Moreover, the proposed method has been found to be applicable beyond a single plant disease. Given its reliance on hyperspectral imaging and advanced feature extraction techniques, it can be adapted for detecting a wide range of plant diseases across different species. By training models on spectral data from various plant–pathogen interactions, this method can be extended to identify fungal, bacterial, and viral diseases, broadening its applicability in agriculture. Overall, this research contributes to the advancement of precision agriculture, ensuring that even small-scale farmers can benefit from cutting-edge plant disease detection technologies.

While detailed spectral analysis of the differences between healthy and diseased plants can provide valuable insights, the primary focus of this study is not on characterizing spectral signatures but on developing a rapid and early disease detection method. This method utilizes few-shot learning combined with EfficientNet feature extraction and Manhattan distance metrics to achieve practical and scalable detection. Therefore, detailed plots of average reflectance curves and spectral feature interpretation are beyond the current scope but represent an important direction for future research.

Compared to previous research that heavily relies on large RGB datasets or computationally intensive deep learning methods, the proposed approach offers several key advantages. First, by utilizing hyperspectral imaging combined with few-shot learning, our method reduces dependency on extensive labeled datasets, making it more practical for real-world agricultural applications where data annotation is expensive and time-consuming. Second, while earlier studies often focused on single disease types or required high-resolution imagery with large computational demands, our approach achieves accurate multi-class classification of four fungal diseases using a compact dataset and a lightweight architecture (EfficientNet). This demonstrates that high diagnostic performance can be obtained with significantly fewer resources, thus increasing the feasibility of real-time, cost-effective deployment in precision agriculture. Researchers found that Convolutional Neural Networks (CNNs) are effective in automating plant disease detection, supporting crop health monitoring. However, their limited interpretability restricts their practical use in real-world agriculture. To address this, advanced architectures like ResNet-50 and EfficientNet, combined with attention mechanisms, were explored. These models improve accuracy by optimizing network structure and enhance interpretability by focusing on disease-specific image regions. Experiments on the PlantVillage dataset showed that while basic CNNs achieved 46.69% accuracy, ResNet-50 and EfficientNet reached 63.79% and 98.27%, respectively.

A limitation of this study is that the proposed framework has not yet been tested across different field conditions. While the experimental results demonstrate the method’s feasibility in controlled settings, real-world deployment involves greater variability in environmental conditions, sensor types, crop varieties, and disease expression. These factors can significantly influence model performance. To improve robustness and generalizability, future work should focus on testing the framework in diverse geographical locations and under various agronomic conditions. Incorporating larger and more heterogeneous field-based datasets, along with domain adaptation or transfer learning techniques, will be crucial for enabling broader applicability.

## 5. Real-Time Disease Detection in Agriculture Using IoT and Few-Shot Learning

The integration of the IoT in agriculture has revolutionized the way farmers monitor and manage their crops, particularly for early disease detection. IoT enables the connection of various devices such as sensors, cameras, and drones to monitor real-time environmental conditions like temperature, humidity, and soil moisture. These devices collect crucial data, which is then processed by machine learning models to detect diseases before they spread. The power of IoT lies not only in data collection but also in its ability to share that data across networks, allowing farmers, agricultural experts, and other stakeholders to be alerted instantly. This real-time communication is vital for early disease detection, ensuring that preventative measures can be implemented quickly, potentially saving entire crops from devastating diseases [40]. Few-shot learning (FSL) enables plant disease detection models to adapt with minimal labeled data, overcoming the challenge of collecting large datasets. This makes disease diagnosis more efficient, accessible, and cost-effective, especially in resource-limited areas [41].

By integrating IoT with few-shot learning, farmers can receive timely alerts whenever a disease is detected, enabling them to take immediate action. The IoT system triggers alarms or notifications when disease symptoms are recognized, sending real-time information directly to the farmer’s mobile device or a centralized portal. This system can also share alerts with neighboring farms, promoting community-wide disease management efforts. The early detection facilitated by IoT and FSL significantly reduces the risk of diseases spreading, as timely intervention is key to controlling outbreaks. Furthermore, these alerts provide valuable insights into disease severity, allowing farmers to understand the exact nature of the threat and take informed actions, whether it be applying pesticides, adjusting irrigation, or removing infected plants [42].

One of the key advantages of this technological integration is its ability to provide early disease detection. While hyperspectral imaging systems are not low-cost, they can detect diseases much earlier than traditional methods. In contrast, traditional imaging often requires expensive equipment and complex software, which can be inaccessible to small-scale farmers [43]. However, hyperspectral imaging systems can capture detailed spectral data, enabling deep learning models to identify subtle spectral changes associated with disease progression. Unlike traditional methods that require manual feature extraction, deep learning models automatically extract relevant features from raw images, reducing both costs and complexity. By eliminating the need for manual intervention and allowing real-time processing, these systems become not only cost-effective in the long term but also scalable and easy to deploy across various farms [44,45].

The combination of IoT, few-shot learning, low-cost imaging, and deep feature extraction provides farmers with a powerful tool set for early disease detection and management. This innovative approach simplifies disease diagnosis while making it more affordable for farmers of all sizes. It is particularly valuable in regions where resources are scarce and traditional disease management methods may not be viable. Moreover, the IoT-based alarm system enables farmers to act swiftly, preventing the spread of disease and minimizing crop loss. As these technologies continue to evolve, they will create new opportunities for global agriculture, making it possible for farmers to monitor and protect their crops more efficiently and sustainably. Through shared knowledge, collaboration, and technological innovation, the agricultural community can work together to mitigate the impact of plant diseases and improve overall productivity [46,47].

In summary, the integration of IoT systems, few-shot learning models, and low-cost imaging technologies offers a transformative solution to early plant disease detection. By improving the accuracy and speed of diagnosis, these technologies not only enable farmers to respond quickly but also make the process more affordable and accessible. With the power of real-time data sharing and community-based disease management, this approach holds the potential to revolutionize agriculture, making it more efficient, sustainable, and resilient in the face of emerging plant diseases. As more farmers adopt these technologies, the industry will see a significant reduction in crop loss and a boost in agricultural productivity.

## 6. Conclusions

This study presents a hybrid model that integrates deep learning and hyperspectral imaging to address the critical challenge of early detection of fungal diseases in tomatoes. By combining automated feature extraction with the Manhattan distance-based classification, the model effectively operates under few-shot learning conditions, requiring minimal training data while maintaining robust performance. The use of hyperspectral imaging enables continuous, non-invasive monitoring of plant health, capturing subtle spectral variations that signal disease progression well before visual symptoms become apparent.

The findings demonstrate that the timing of disease detection is pivotal. Routine visual assessments indicated that *A. alternata* and *B. cinerea* became visibly symptomatic by Day 5 post inoculation, while *A. solani* and *F. oxysporum* exhibited no symptoms until Day 7. In contrast, the proposed hybrid model was able to detect *A. alternata* and *B. cinerea* reliably by Day 3, two days prior to symptom visibility, highlighting its capacity for pre-symptomatic detection. *A. solani* and *F. oxysporum* required more time, reaching reliable detection levels by Day 5, still preceding the onset of visible symptoms. The gradual increase in detection accuracy over time highlights the strength of few-shot learning frameworks in adapting to limited data environments, particularly when paired with temporal imaging data. These results also emphasize the need for disease-specific timing strategies, as each pathogen exhibits unique progression dynamics.

By identifying the earliest reliable detection points for each disease, this research contributes actionable insights for early intervention, ultimately enabling more precise and timely disease management. The proposed approach is scalable, cost-effective, and well suited for real-world agricultural settings, offering a valuable tool to reduce crop losses, enhance productivity, and support sustainable farming practices. As a result, this model not only advances the field of plant phenotyping and digital agriculture but also empowers growers with timely, data-driven decisions for disease control.

## Figures and Tables

**Table 1:** Tomato fungal disease detection across different days post inoculation using cosine similarity.

		Day 1	Day 3	Day 5	Day 7
5-Way 1-Shot Learning	*A. alternata*	11	18.98	32.11	43.56
	*A. solani*	6.35	12.09	20.03	36.28
	*B. cinerea*	8.6	22	42.08	53
	*F. oxysporum*	7.15	16.44	39.7	50.51
	Healthy	61.33	60.18	61.9	61.27
5-Way 3-Shot Learning	*A. alternata*	18.86	41.5	65.99	76.82
	*A. solani*	9.8	27.11	50.43	63.17
	*B. cinerea*	15.9	54.3	70.22	85.2
	*F. oxysporum*	13.6	25.02	58.39	71.66
	Healthy	90.13	89.51	90.02	90.5

## Data Availability

The data supporting the results of this study are available from the corresponding author upon reasonable request.
